# Leprosy and Leper Houses

**Published:** 1889-11-30

**Authors:** 


### Leprosy and Leper Houses.

There was a large attendance at the second general meeting of the Hospitals Association of the present session, held in the board-room of King's College Hospital on Wednesday, 20th inst.; Prebendary Wace, D.D., in the chair. Present: Lieut.-General R. H. Keatinge, V.C.; Richard Horton Smith, Q.C. (governor of King's College Hospital); Theobald Ringer (Deputy Surgeon-General, Cheltenham); Dr. J. C. Steele; Keith D. Young; P. Wigram; Dr. H. B. Dow (St. John's Hospital for Skin); Dr. P. S. Abraham, M.A., B.Sc.; Dr. Walter Pocock (hon. surgeon London Skin Hospital); P. Michelli; T. Ryan and Mrs. Ryan; H. Howgrave Graham; W. M. Hughes (Cancer); R. A. Owthwaite; Horace A. Gifford (secretary London Skin Hospital); Rev. N. Bromley, and several matrons and nurses.

Sir Wm. Moore, K.C.I.E. (late Surgeon-General Bombay Staff), delivered a lecture on "Leprosy and Leper Houses." The lecturer remarked that the subject was a most important one, both from a medical and from a social point of view, and the disease of leprosy demanded the attention of all scientific associations. The public fully supported the Prince of Wales in his efforts on behalf of the memorial for Father Damien. But there was not the same unanimity in favour of the establishment of a leper ward in London, it being urged, on the one hand, that the ward would be constantly empty, and, on the other, that lepers would flock to it and that it would become a centre of contagion. Lepers were always to be found in England, but that they would flock to a ward in London, if it were established, might be questioned. Very few people knew what leprosy was. It was not a very painful disease, and while it was hereditary, it was seldom contagious. The prevalence of the disease in India had been greatly exaggerated. The fact that mendicant lepers were frequently seen in comparatively large numbers in Indian towns was responsible for this. The idea that nothing was done for lepers in that country was also erroneous. There were, on the contrary, various and efficient organisations for the amelioration of their lot. In a discussion which followed—

Dr. Abraham said he had listened with the deepest interest to Sir William Moore's lecture, but begged leave to differ from his views on some points. Sir Wm. Moore did not wish to be regarded as an authority, but he (Dr. Abraham) thought he was a very great authority on leprosy. He thought the lecturer was right in holding that there was no danger in establishing a leper ward in this country. But some other things were said which several medical men would hardly agree with. For instance, that the leprosy of cold climates was not the same kind of leprosy as the leprosy in warmer climates. Those who had seen it in both places could not see any difference whatever. It ran the same course, and remedies were precisely the same. There was strong testimony that leprosy in Norway was the same as leprosy in the South of Europe. Most medical men might hold with Sir Wm. Moore that leprosy was hereditary, but recently some change appeared to be coming in this view. The probability was, according to recent opinion, that this element had not so much to do with the disease as the element of intercourse between person and person. The fact of several lepers being in one family did not establish the hereditary principle, but the various members were more likely to be in contact together, and the explanation might be found in that fact. A great authority in Norway now had been in North America a short time ago, and enquired as to what had become of several Norwegian lepers who had gone there. Several of them had married. He could trace them to the third and fourth generation, and in no case had their progeny become lepers, and this authority held there could be no stronger evidence against heredity. The fish diet theory was not exactly a new one, but had been resuscitated recently by Sir John Hutchinson. The view was propounded in old books, but did anyone now agree with it? He had an opportunity of speaking on this subject to the doctor of the Shah of Persia, who had told some of them that there was very little leprosy in the lowlands of Persia, and that at Teheran he did not know of a single case, but in the mountains far away from the coast there was a good deal of leprosy. There people were cut off from fish diet by their surroundings, the want of means for conveying it from the lowlands, and by their methods. The view taken by Sir William Moore, that leprosy was not known in the South Sea Islands before 1852, had been combated on several occasions. At that time the case of Chinamen there came prominently forward, but in recent reports it was accepted that his was not the first case. No doubt since 1860 the disease had enormously increased.

Prebendary Wace, on behalf of the meeting, thanked the lecturer for his address, interesting in many ways, and not merely from a medical point of view. He could not but feel interested in reference to the prevalence in Europe of what was believed to be proper leprosy in the middle ages. One of the ways in which the Franciscan Order acquired its influence was through the devotion its members displayed in the treatment of what was then believed to be this frightful disease. Although it was easy to understand what Sir William Moore had said about the inaccurate diagnosis of disease, yet the horrible descriptions they had n the accounts of the Franciscan Order could leave no doubt, he thought, that leprosy in its worst form was a terrible scourge in Europe at that time. He had in his hand the "Monumenta Franciscana" (Roll's Series of Public Documents), edited by Prof. Brewer. These were Latin documents, but there were some hundred pages of preface, which outlined the documents and gave some notion of the condition of Europe in the thirteenth century. The unsanitary condition of dwellings was shown, the narrowness and intricacy of the streets, as a protection against the armed night, but a means of decimating the population, the lazy ditches and stagnant ponds, the stifling chamber of the open booth of the artisan. St. Francis had once turned away from a leper, and asked as a penance that he should eat out of the same dish with his Christian brother. The members of his order bound themselves to the personal care and attendance of lepers, washing them, and performing other personal services towards them. Thus they had won the confidence of the common people. It was curious to think that St. James's Palace was the remains of an old lazar house. In the thirteenth century the whole of what was now the park was an immense swamp, and there was a lazar house dedicated to St. James. Gradually brought into cultivation, the place became what it was at present. The care of lepers had been continuous, no doubt, with certain lapses, for centuries throughout Christian Europe, and they might say at no time entirely disregarded. But it was the privilege of some persons, at particular times, by some act of self-sacrifice, to draw fresh attention to the subject. That had been the privilege of Father Damien. In paying all honour to him, they were not to forget the others who had acted in a precisely similar manner. In 1882, the British Government of the Cape of Good Hope placed the lepers there in charge of the Moravian Church. The missionaries and their wives, for more than forty years, continued to devote themselves to that work. They must be thankful that renewed attention had been called to such a terrible scourge of humanity. There seemed every reason to believe that it could be indefinitely warded off—and, they hoped, banished entirely—by proper sanitary conditions and proper food. They must express their sense of the way in which the Prince of Wales, as usual, was devoting himself to an urgent and permanent want, and endeavouring to promote the establishment of a leper fund, towards which some steps had already been taken, and still more active steps, he believed, were to be taken in future.

Sir Wm. Moore, in thanking the meeting for their reception of his paper, said he did not maintain that there was any particular difference in the disease in cold and hot climates, but gave his impression that it was not so inveterate in cold as in hot climates. The same remark might be made about various other diseases common to both. It was curious that the Chinese thought a visit to the cold north the best way to remove it. There was a want of stamina, less vital power, in hot countries to resist it. In reference to heredity, in the case of the Norwegian lepers whose children in North America were not lepers, the change of climate might have had something to do with it. They got out of an endemic area, he supposed. He could not give up his views about heredity, because in his own experience in the examination of lepers he came to the conclusion that thirty per cent. of them acknowledged a leprous taint in the family. Again, leprosy abounded where people never saw fish, and did not know what it was. As to the South Sea Islands, he had given the time when the first leper was discovered there, but the disease might have existed in the place from time immemorial. He stated, in answer to Mr. Howgrave Graham, that the Bill in reference to lepers which the Government of India had recently brought forward and passed, did not contemplate going further than he had mentioned in his address. It proposed to confer on district magistrates the power to order the arrest of any leper found wandering or begging, and who was without the means of subsistence. Legislation at present had only gone so far, and it was as far as he had advised that it should go at present. He had received only yesterday (and not in time to go through it very minutely) a report of a very long debate on this Bill by the members of the Bombay Medical and Physical Society. When he said that this society was composed of native and European practitioners, and of civil and military servants, they would agree that perhaps there was no body of men better able to give an opinion on the subject. They all, with one exception, agreed with the principles of this Bill, but they said there ought to have been another clause to prevent lepers from being engaged in laundry work or having any connection with the sale of edibles, and he thought that would have been a good additional clause. They all ridiculed the idea of any scare on the subject of leprosy, and said there was no reason to suppose that leprosy was more prevalent than it used to be, but that a desire for alms brought a great many lepers to Bombay from other parts of India. What was somewhat unusual in such societies, the members were almost unanimous in the matter.

Dr. Steele proposed, and Gen. Keatinge seconded, a vote of thanks to the chairman for presiding, and to the chairman and committee of King's College Hospital for the use of its board-room for the present meeting, which was carried unanimously.

